# Altered Circadian Rhythms and Breast Cancer: From the Human to the Molecular Level

**DOI:** 10.3389/fendo.2018.00219

**Published:** 2018-05-04

**Authors:** Hui-Hsien Lin, Michelle E. Farkas

**Affiliations:** Department of Chemistry, University of Massachusetts, Amherst, MA, United States

**Keywords:** altered circadian rhythms, shift work, breast cancer, molecular mechanism, hormone pathways, small molecule modulators

## Abstract

Circadian clocks are fundamental, time-tracking systems that allow organisms to adapt to the appropriate time of day and drive many physiological and cellular processes. Altered circadian rhythms can result from night-shift work, chronic jet lag, exposure to bright lights at night, or other conditioning, and have been shown to lead to increased likelihood of cancer, metabolic and cardiovascular diseases, and immune dysregulation. In cases of cancer, worse patient prognoses and drug resistance during treatment have also been observed. Breast, colon, prostate, lung, and ovarian cancers and hepatocellular carcinoma have all been linked in one way or another with altered circadian rhythms. Critical elements at the molecular level of the circadian system have been associated with cancer, but there have been fairly few studies in this regard. In this mini-review, we specifically focus on the role of altered circadian rhythms in breast cancer, providing an overview of studies performed at the epidemiological level through assessments made in animal and cellular models of the disease. We also address the disparities present among studies that take into account the rhythmicity of core clock and other proteins, and those which do not, and offer insights to the use of small molecules for studying the connections between circadian rhythms and cancer. This article will provide the reader with a concise, but thorough account of the research landscape as it pertains to altered circadian rhythms and breast cancer.

## Introduction

It was first reported in the 1960s that circadian rhythm disruptions can lead to an increased likelihood of mammary tumor development, and that circadian genes may act as tumor suppressors (1). In previous decades, studies have suggested that alterations to circadian rhythms also accelerate breast epithelial stem-cell proliferation, induce mammary-gland development, and increase the formation of spontaneous breast tumors in mammals (2, 3). Disruptions to circadian rhythms in humans have also been associated with the development of several other cancer types, including prostate (4), endometrial (5), colon (6), lung (7), and ovarian cancers (8) and hepatocellular carcinoma (9). In addition, the rhythmic control of cell fate is believed to affect cancer therapies: the efficacy and/or toxicity of radiotherapy and antitumor therapeutics have been shown to be dependent on the timing of dose administration (10, 11). Thus, understanding the link between biological rhythms and cancers can both assist in the development of new treatments, and in optimization of current therapies.

In mammals, the molecular circadian clock can be divided into three components: input pathways, the central pacemaker, and output pathways. The input pathways transmit information from environmental cues (e.g., light) to the central pacemaker, which is located in the suprachiasmatic nucleus (SCN) of the hypothalamus (12). Within the SCN, multiple single-cell circadian oscillators are synchronized to generate daily circadian outputs (13). Output pathways convert the commands from the central pacemaker into circadian oscillations, which regulate physiological and behavioral functions in peripheral organs and tissues (14).

Circadian oscillations are mainly generated through two transcriptional/translational feedback loops (TTFLs) (15). The core loop involves four core clock genes: Circadian Locomotor Output Cycles Kaput (*CLOCK)* (16) and brain and muscle Arnt-like protein 1 (*BMAL1*) (17), which are the activators; and Period (*PER1, PER2*, and *PER3*) (18) and Cryptochrome (*CRY1* and *CRY2*) (19), which are the repressors. In the morning, the *CLOCK*:*BMAL1* heterodimer binds to an E-box DNA promoter, activating the transcription of *PER, CRY*, and other clock controlled genes. Late in the day, PER and CRY proteins dimerize and translocate from the cytoplasm to the nucleus, where they associate with the *CLOCK*:*BMAL1* complex and suppress its transcriptional activity at the E-box site (20). The suppression of *CLOCK*:*BMAL1* is released through the degradation of PER and CRY by ubiquitin-dependent pathways (21, 22) and casein kinases (CKIδ and CKIε) (23), which also control the timing of PER and CRY’s entrance to the nucleus. After PER and CRY are degraded, the cycle begins again with ~24 h periodicity.

The secondary TTFL is mainly driven by transcriptional activation of the retinoid-related orphan receptors (RORs a, b, c) and repression of REV-ERBα/REV-ERBβ (24). To drive the rhythmic oscillation of BMAL1, REV-ERBα binds to the ROR elements in the *BMAL1* promoter, suppressing *BMAL1* transcription. Conversely, RORa and RORb activate *BMAL1* expression (25, 26). The cooperation between the two TTFLs and other kinases and phosphatases, which are critical for regulating period, phase, and amplitude of oscillations, provides robustness against environmental perturbations. This network also helps to maintain accurate circadian timing and adjust phase delays to align with local physiology (27).

## Epidemiological Evidence of Altered Clocks’ Effects on Cancer

Lifestyles have dramatically changed since the invention of the light bulb in 1879. Since then, the daily activities of humans have expanded into the night, including “night-shift” occupations (28). According to the U.S. Bureau of Labor Statistics, in 2016, the majority of the employed population worked in the service industry (80.3%), including health care, social assistance, and transportation, followed by manufacturing (7.9%) (29)—areas with high proportions of shift work. Another report published in 2015 found that about 17–24% of the workforce in United States was assigned to irregular or on-call work schedules, including night and rotating shifts (30). These types of schedules can lead to disruption of the sleep–wake cycle and circadian time organization, in addition to exposure to light at night (LAN) for long periods of time (31, 32). Perturbations to sleep and circadian rhythms can cause metabolic changes (33) and immune suppression (34), which can lead to various health problems, including diabetes (35), obesity (36), and cardiovascular disease (37), in addition to cancer (38). As a result, the International Agency for Research on Cancer has classified “shift-work that involves circadian disruption” as a “potential carcinogenic to humans (Group 2A)” (39).

While debated in some instances, epidemiological studies have provided evidence to support the association between shift work and cancer risk (40, 41). Independent cohort studies of night workers and shift workers have observed increased incidence of breast (42), prostate (4), colon (43), and endometrial epithelial malignancies (44) and non-Hodgkin’s lymphoma (45), with risk further increased among individuals who have spent more hours and years working at night (42, 46). A case control study in Western Australia found that there was a 22% increase in breast cancer incidence among those who worked between midnight and 5:00 a.m. (47). Another study in France showed that there was a significant association (OR = 1.95) between breast cancer and women who worked night shifts for more than 4 years before their first full-term pregnancy. At that time their mammary-gland cells were found to be incompletely differentiated, making them more susceptible to circadian disruption effects (48). While it is difficult to eliminate shift work from society, there are some aspects that can be modified, which may decrease the risk of developing adverse health effects. To further understand the contributions of shift work to pathological development, extensive animal and cellular experiments have yielded proposed molecular mechanisms, which will be discussed in Section “Molecular Studies of Circadian Clocks and Breast Cancer.”

Jet lag is another environmental factor associated with altered circadian rhythms and higher incidence of cancers (49). Jet lag (or circadian desynchrony) is a sleep disorder arising from the mismatch between internal body clocks and the environmental light/dark cycle. This condition is typically the result of travel through multiple time zones over a short period of time (50). An early study in Finland showed that flight attendants have significantly higher incidence of breast cancer (81.2/100,000) compared with the general female population (57.4/100,000) (51). A later, follow-up assessment strongly suggested that the increased cancer incidence was related to disruption of sleep rhythms, caused by excess exposure of light during normal sleeping hours, resulting in melatonin dysregulation (52). In addition, a recent study published in 2017, which focused on the effect of exposure to LAN in the United States, showed that there was a 14% increased risk of breast cancer in the highest LAN compared with the lowest LAN (53). Similar results were reported in Israel, where there was a 73% higher incidence of breast cancer in communities with the highest LAN than lowest LAN, across 147 communities (54). All of these epidemiological studies have strongly indicated that the disruption of circadian rhythms contributes to cancer risk.

## Molecular Studies of Circadian Clocks and Breast Cancer

The functions of clock genes in each tissue are unique and show specific oscillation patterns (55). Their expression and regulation play important roles in breast biology. It has been shown that the core clock genes exhibit different mRNA expression patterns during mammary-gland development and differentiation in mice (56). Among 14,070 tested genes in human epithelial cells, 1,029 genes showed rhythmic oscillations during lactation. The expression patterns of these genes can be clustered into two groups, one high in the morning and another in the evening, indicating that the expressions change with a period of 24 h (57). Not only are the expression levels of clock genes variable, but they are affected by different developmental stages of breast tissue, and the extracellular microenvironment (58). Thus, it is posited that disruption of clock genes can affect normal breast biology and induce or affect cancerous development.

Breast cancer is heterogeneous and can be classified into subtypes based on histology, tumor grade, lymph node status, and the presence of specific biomarkers (59). The three markers generally used in characterization are estrogen receptor (ER), human epidermal growth factor receptor 2 (HER2), and progesterone receptor (PR) (60, 61). Based on marker status, breast cancer can be grouped into at least four subtypes: luminal A (ER^+^, PR^+/−^, HER2^−^), luminal B (ER^+^, PR^+/−^, HER2^+^), HER2 (ER^−^, PR^−^, HER2^+^), and Basal (ER^−^, PR^−^, HER2^−^) (62, 63). Basal tumors are typically difficult to treat and have poor prognoses. Because they lack ER, PR, and HER2, they are sometimes referred to as “triple-negative.”

**Figure 1:**
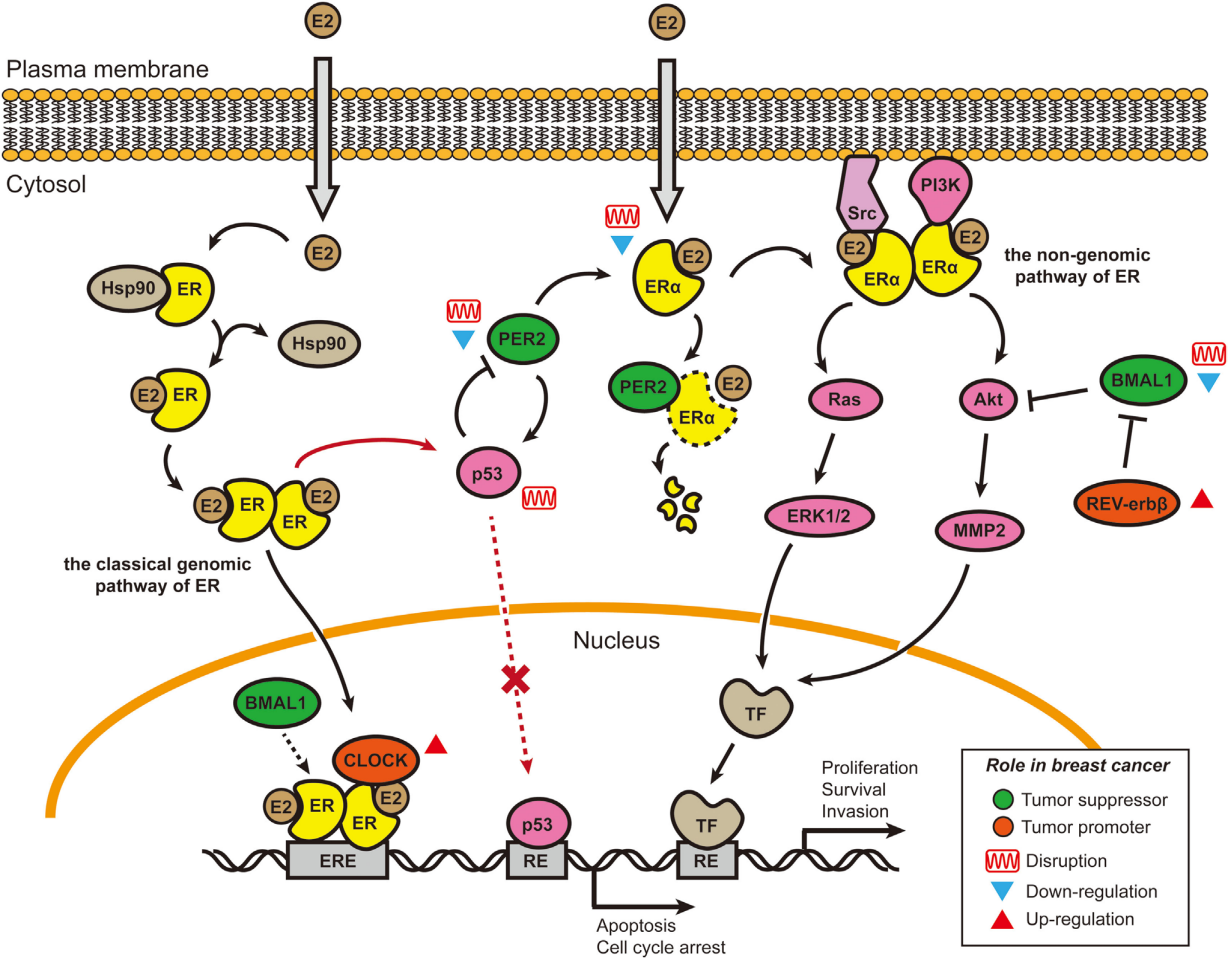
Cross talk between E2–estrogen receptor-α (ERα) signaling pathways and circadian rhythms in breast cancer. Two of the four estrogen signaling pathways involve E2 stimulation and are shown here (65). In the classical genomic pathway, E2-bound estrogen receptor (ER) (either ERα or ERβ) dimerizes, changes conformation, translocates to the nucleus, and binds to the estrogen response elements (EREs). After binding to the EREs, the E2–ER complex recruits other co-activators, including Circadian Locomotor Output Cycles Kaput (CLOCK) and possibly brain and muscle Arnt-like protein 1 (BMAL1) (74), to initiate the transcription of target genes. CLOCK overexpression in breast tumors and promotion of tumor cell proliferation may be caused by co-activation with E2–ER complexes (77, 78). In the non-genomic pathway, E2–ERα complexes accumulate near the membrane and then recruit protein kinases [Src and phosphoinositide 3-kinase (PI3K)] to activate signaling cascades (Akt and Ras/MAPK). BMAL1 has been shown to suppress the Akt/MMP2 pathway and further inhibit cancer cell invasion (79). BMAL1 can suppress cancers, and its expression is downregulated or disrupted in various breast cancer cell lines (67, 80–83). By contrast, REV-ERBβ (a repressor in the secondary transcriptional/translational feedback loop) is generally overexpressed in breast tumor samples; its protective function can allow cancer cells to develop chemotherapy resistance (84). *PER2* is a direct ERα target gene and can bind to ERα and cause its degradation. In ERα-positive breast cancer cells, both PER2 and ERα lose their circadian oscillations, the underlying mechanism of which is not well understood. The cancer suppressor p53 can directly bind to the *PER2* promoter and inhibit its transcriptional activity (85). E2–ER complexes can block the induction of proapoptotic p53 target genes by binding to p53 protein in ER-positive breast cancer cells, thus helping cancer cells avoid apoptosis (73). Re-introduction of PER2 into the ER-positive breast cancer cells can induce p53 expression (72). Abbreviations: TF, transcriptional factor; RE, response element; E2, 17 β-estradiol.

The disruption of nuclear hormone levels and signaling has also been posited to alter circadian rhythms, drawing another connection between rhythms and breast cancer (64). The estrogen receptor-α (ERα) signaling pathway (65) has been linked to the disruption of PER2 in breast cancer (Figure 1) (66, 67). It is known that *PER2* is a direct transcriptional target of ERα and its expression is inducible by 17 β-estradiol (E2) simulation (64, 68). In normal human breast epithelial cells, both *ER*α and *PER2* show rhythmic oscillations. The ubiquitous presence or absence of clock proteins has been predominantly used to investigate the relationship between circadian rhythms and breast cancer development (Table 1) (69–71). Knockdown of either *PER2* or *ER*α results in aberrant circadian oscillations of *ER*α, *PER2, BMAL1*, and *RARA* (another direct ERα target gene) and affects breast acinus structures (66). It was first reported in 2007 that suppression of *PER2* leads to ERα stabilization, and conversely, overexpression of *PER2* in breast cancer cells significantly inhibited cell growth and promoted apoptosis (64, 72). This work was corroborated by showing that complete loss of *PER2* mRNA oscillations occurred only in ERα-positive breast cancer cells, while ERα-negative breast cancer cells retained partially rhythmic oscillations (66, 67). In mice, downregulation of *PER2* enhanced breast tumor growth, leading to further enhancement of amplitude and phase delay (70). All of these studies have suggested that the expression of clock genes may be disrupted by hormone levels and their signaling circuits (Figure 1) (73, 74). In addition, genome-wide DNA methylation profiling has shown that PER1 is significantly hypomethylated in ER^+^/PR^+^ breast cancer tissues (75). A separate study also showed that PER1, 2, and 3 exhibited deviant protein expressions in 55 resected breast cancer tissue sections, when compared with adjacent non-cancerous tissue samples. These fluctuations may be the result of methylation of the *PER* promoter (76). However, the detailed mechanisms of how hormone signaling affects circadian clocks and *vice versa* are still unclear.

**Table 1:** Roles of clock genes in breast cancer development.

Circadian genes	Experimental approaches	Phenotype	Possible mechanism	Reference
*CLOCK*	Immunohistochemical assay(s) and qRT-PCR	Overexpressed in breast cancer cells; low expression in healthy breast tissue	Increased methylation in *CLOCK* promoter decreases breast cancer risk	(77, 78)

	Knockdown(s)	Reduced cell proliferation; downregulation of cancer-associated genes (CCL5, BDKRB2, and SP100)	E2–estrogen receptor (ER) pathway may couple to the circadian machinery due to presence of estrogen response element in the *CLOCK* promoter	(77, 78)

*BMAL1*	qRT-PCR	Disrupted mRNA expressions in breast cancer cells	Not clear	(67, 80–83)

	Knockdown(s)	Promoted cancer cell proliferation and invasion *in vitro* and tumor growth *in vivo*	Antagonized *Bcl-w* oncogene, which can activate phosphoinositide 3-kinase (PI3K)/Akt/MMP2 pathway; effects on *p53* and *c-myc* are cell-type specific	(71, 79)

*PER1, 2, and 3*	Immunohistochemical assay(s) and qRT-PCR	Downregulated in ER-positive breast cancer cells	Methylation in *PER* promoter in ER^+^/PR^+^ breast cancer tissues	(70, 75, 76)

	Knockdown(s)	Aberrant circadian oscillation of other clock genes; enhanced tumor growth *in vivo*; changed the structure of breast acinus	Coupling with E2–ER pathway and p53 pathway	(66)

	Overexpression	Significantly inhibited cell growth and promoted apoptosis	Inhibit the activation of ER and p53 target genes	(64, 72)

*CRY1 and 2*	qRT-PCR	Disrupted mRNA expressions in breast cancer cells	Not clear	(67, 80)

*REV-ERB*α	RNAi screen	Co-expression in *ERBB2*-positive breast tumors (HER2^+^ subtype)	Upregulating several genes that are involved in *de novo* fatty acid synthesis, which further enhance the energy production for survival	(86)

*REV-ERB*β	Overexpression	Protect tumor cells against chemotherapy	Not clear	(84)

BMAL1 has also been proposed to act as a tumor suppressor. In separate studies performed in lung cancer and glioma cells, knockdown of *BMAL1* promoted cancer cell proliferation, invasion, and tumor growth, while its overexpression reduced cellular invasiveness (71, 79). Effects occurred in a p53-independent manner (*p53* expression was decreased in all *BMAL1* knockdowns) and were accompanied by activation of the phosphoinositide 3-kinase (PI3K)–Akt–MMP-2 signaling pathway (79). While these studies used other cancer models to study the role of *BMAL1*, the findings are likely relevant to breast cancer. *p53* mutations in breast cancer are relatively frequent (~20%) (85, 87), and the PI3K/Akt pathway is commonly affected (~70%) (88). However, the same study found that p21 (a p53 target protein) and c-myc exhibited different expression levels in various *BMAL1*-knockdown colon cancer cells, indicating that the relationships among BMAL1, p21, and c-myc are probably cell-type specific (71).

By contrast, CLOCK has been indicated as a tumor driver. Healthy breast patient tissues showed lower CLOCK expression than breast tumor tissues (77, 78). Knockdown of *CLOCK* resulted in attenuation of breast cancer proliferation (77) and downregulation of several cancer-associated genes, including ones related to breast tumor progression and metastasis initiation, such as *CCL5, BDKRB2*, and *SP100* (78). Furthermore, increased methylation in the promoter region of *CLOCK* has been associated with decreased breast cancer risk (78). While these studies provide valuable insight to the involvement of clock proteins in breast cancer development, most of these experiments do not account for the dynamic nature of circadian rhythms, and the fact that they may be altered but not abolished with human behavior and disease.

More recently, a number of *in vitro* studies have investigated clock gene expression profiles in a time-dependent manner in various breast cancer cell lines, including: MCF7 and T47D (luminal A subtype); HCC-1954 (HER2 positive subtype); MCF10A and MDA-MB-231 (basal-like subtype), and others (67, 80–83). Intrinsic circadian oscillations in cultured cells can be entrained through treatment with high concentrations of serum to serum starved cells (89), or by chemical induction of signaling pathways, such as protein kinase A (*via* forskolin) (90) or the glucocorticoid receptor (*via* dexamethasone) (91). After entrainment, the expression patterns of clock genes, including *BMAL1, CLOCK, PER1, PER2, CRY1*, and *CRY2*, have largely been analyzed through quantitative real-time PCR, with conflicting results. While some studies revealed rhythmic gene expression in all breast cancer cell lines (67, 82), others did not (80, 83). Major factors contributing to the discrepancies were likely non-uniform cell culture and synchronization methods (i.e., varied serum depletion times before serum shock), which may affect dampening rates over time, due to loss of synchronicity. In addition, the short-term data collection (typically ≤48 h) and insufficient numbers of data points (generally ≥4 h intervals) utilized in these studies are generally insufficient to yield good statistical curve fittings (92, 93), contributing to inaccurate analysis of rhythmic oscillations. However, within each study, it is apparent that oscillations of clock proteins vary across different breast cancer cell models. Application of luciferase reporters and fluorescent proteins (e.g., GFP) can provide better resolution for long-term tracking of circadian oscillations in synchronized cells (14). However, cancer cells can be heterogeneous even in culture conditions (94). Future work should focus on real-time analysis at the single-cell level to reveal how circadian rhythms are involved, disrupted, and deviate from one another in breast cancer. Furthermore, posttranscriptional and -translational modifications to core circadian clock components should also be taken into consideration (95), since many malignant transformations occur posttranscriptionally.

## Circadian Chronotherapy and Cancer Treatment

Nearly, all metabolic functions are regulated in a circadian manner: food intake, digestion, detoxification, breakdown, and storage of sugars and fats (96–98). When organs are exposed to xenobiotics (e.g., drugs or environmental toxicants), they undergo classical absorption, distribution, metabolism, and elimination processes, which are all regulated by circadian clocks (11). Hence, accounting for circadian rhythms in the development of treatments and dosing regimens has the potential to improve disease outcomes. Two recent studies reported the effects of chemotherapy on circadian rhythms in patients with metastatic colorectal cancer (99, 100). It was found that chemotherapy-induced disruption was observed in approximately 50% of the patients and was correlated with shortened overall survival rate. Eliminating this perturbation has been suggested to reduce toxicity and enhance efficacy of chemotherapy.

Recently, compounds that specifically target clock components and/or modulate its oscillations have received a great deal of attention (101). There are many advantages to the usage of small molecules in studies of circadian-related diseases: (1) they can help us to better understand the molecular circadian network; (2) they can serve as lead structures for developing drugs; and (3) unlike genetic approaches, which can result in immutable modifications, small molecules can be used in reversible, time- and dose-dependent manners (102, 103). One common example is the amelioration of jet lag *via* use of the hormone melatonin (104, 105). A double-blind trial showed that melatonin can significantly reduce jet lag and sleep disturbance in an international cabin crew (106). Small molecules can also be used to modify circadian rhythm periods to minimize chronodisruption resulting from shift work. Since the entrained phase is associated with circadian period, the period modification should change the preferred phase of behavior (107). Amplitude enhancement has also been shown to combat metabolic syndromes (108), reverse age-related effects (109), and protect against psychiatric diseases (110).

Small molecules have been used to elucidate the connections between circadian rhythms and breast cancer, for example the role of *REV-ERBs* in the HER2^+^ subtype (111). The *NR1D1* (REV-ERBα coding gene) is connected to *ERBB2* (HER2 coding gene) in the 17q12 amplicon, resulting in their co-expression in *ERBB2*-positive breast tumors (86). The same study suggested that REV-ERBα serves as a survival factor for HER2^+^ breast cancer cells. However, more recent work has shown disagreements. By activating REV-ERBs *via* the synthetic agonist SR9011, decreased cell proliferation was observed in various breast cancer cells, independent of their ER or HER2 status (112). Another study found that dual inhibition of REV-ERBβ and autophagy by ARN5187, a novel REV-ERBβ ligand, can induce cytotoxicity in breast cancer cells (84). It was also shown that REV-ERBβ was dominantly expressed in breast tumor samples, while REV-ERBα was the predominant form in normal tissues. Overexpressed REV-ERBβ appeared to result in protection that made tumor cells resistant to chloroquine, a clinically relevant lysosomotropic agent suppressing autophagy. With ARN5187 treatment, REV-ERB-mediated transcription was inhibited. Grimaldi et al. suggested that this compound has the potential to serve as an anticancer agent (84). Although clock modulators alone may not be sufficient to induce anticancer effects, combined treatment with well-established anticancer drugs should enhance their potency and efficacy, and reduce toxicity of the drugs. Characterization of disrupted circadian patterns in various types of cancer can provide clues for the application of clock modulators in combination with anticancer drugs to achieve the best possible therapeutic results.

## Conclusion

Circadian rhythms are essential to the regulation of many physiological and behavioral functions in mammals. Their disruption has been linked to development of many health problems, including breast cancer. This is supported by epidemiological evidence, assessing both shift workers and people exposed to chronic jet lag. The status of core circadian clock components has also been evaluated in cancerous versus healthy tissues, and the significance of these components has been investigated *via* overexpression or deletions. While more recent studies have addressed changes in oscillations across cancer types, investigations at higher resolutions are required to facilitate more thorough analysis. From the work reviewed here, it is clear that circadian rhythms and proto-oncogenes/signaling pathways (e.g., *PI3KCA, p53*, or E2–ER) can both affect one another. However, the molecular mechanisms behind these associations are not well understood, and currently very few studies exist that examine the effects of altered rhythms on oncogenic pathways. Future work should also take advantage of existing technologies (including high-resolution confocal microscopy) (113) to track and analyze dynamic circadian oscillations at the single-cell level. While posttranscriptional and -translational modifications are also critical elements of the puzzle, real-time monitoring of these processes remains difficult to achieve. By increasing knowledge of the molecular mechanisms associated with disrupted clocks in cancer, new therapeutics and adjuvants can be developed with enhanced efficacy against the disease.

## Author Contributions

H-HL wrote this article and generated the graphic and table, with content and editorial revisions from MF.

## Conflict of Interest Statement

The authors declare that the research was conducted in the absence of any commercial or financial relationships that could be construed as a potential conflict of interest. The reviewer EPS and handling Editor declared their shared affiliation.
